# The frugivory network properties of a simplified ecosystem: Birds and plants in a Neotropical periurban park

**DOI:** 10.1002/ece3.6481

**Published:** 2020-08-04

**Authors:** Gabriela I. Salazar‐Rivera, Wesley Dáttilo, Gonzalo Castillo‐Campos, Norma Flores‐Estévez, Brenda Ramírez García, Ernesto Ruelas Inzunza

**Affiliations:** ^1^ Instituto de Biotecnología y Ecología Aplicada Universidad Veracruzana Xalapa Mexico; ^2^ Red de Ecoetología Instituto de Ecología A.C. Xalapa Mexico; ^3^ Red de Ecoetología Instituto de Ecología A.C. Xalapa Mexico; ^4^ Instituto de Investigaciones en Ecosistemas y Sustentabilidad Universidad Nacional Autónoma de México Morelia Mexico

**Keywords:** city, exotic, migratory, mutualism, resident

## Abstract

Frugivory networks exhibit a set of properties characterized by a number of network theory‐derived metrics. Their structures often form deterministic patterns that can be explained by the functional roles of interacting species. Although we know lots about how these networks are organized when ecosystems are in a complete, functional condition, we know much less about how incomplete and simplified networks (such as those found in urban and periurban parks) are organized, which features are maintained, which ones are not, and why. In this paper, we examine the properties of a network between frugivorous birds and plants in a small Neotropical periurban park. We found a frugivory network composed of 29 species of birds and 23 of plants. The main roles in this network are played by four species of generalist birds (three resident, one migratory: *Myiozetetes similis*, *Turdus grayi*, *Chlorospingus flavopectus*, and *Dumetella carolinensis*) and three species of plants (one exotic, two early successional: *Phoenix canariensis*, *Phoradendron* sp., and *Witheringia stramoniifolia*). When compared to reference data from other locations in the Neotropics, species richness is low, one important network‐level metric is maintained (modularity) whereas another one is not (nestedness). Nestedness, a metric associated with network specialists, is a feature this network lacks. Species‐level metrics such as degree, species strength, and module roles, are not maintained. Our work supports modularity as the most pervasive network‐level metric of altered habitats. From a successional point of view, our results suggest that properties revealed by species‐level indices may be developed at a later time, lagging the acquisition of structural elements.

## Graphical abstract

In a Neotropical periurban park, the bird–plant frugivory network maintains only some properties of fully functional networks.

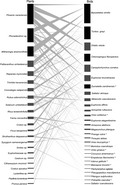

## 1 INTRODUCTION

Interactions between animals and plants are regarded as of paramount importance in the development and maintenance of biodiversity (Andresen, Arroyo‐Rodríguez, & Escobar, 2018; Bascompte & Jordano, 2007; Boucher, 1985; Dáttilo et al., 2016; Thompson, Askew, Grime, Dunnett, & Willis, 2005). Animals consume fruits provided by plants and carry them away from its parent source, often helping to disperse their seeds (Escribano‐Ávila, Lara‐Romero, Heleno, & Traveset, 2018). Birds are known to play a central role in seed dispersal and forest recruitment dynamics (Jordano & Schupp, 2000), and have received lots of attention from researchers, reflected in the largest number of publications among ecological network analyses (Almazán‐Núñez, Arizmendi, Eguiarte, & Corcuera, 2015; Escribano‐Ávila et al., 2018; Gonzalez & Loiselle, 2016; Ramos‐Robles, Andresen, & Díaz‐Castelazo, 2016; Spotswood, Jean‐Yves, & J and Bartolome W, 2012; Thompson et al., 2005).

The relationships between sets of species of birds and plants can form complex patterns of interconnections that have found in network theory a framework to disentangle its structure and function (Bascompte & Jordano, 2014; Carnicer, Jordano, & Melián, 2009). Although many of the most cited complex networks investigations have taken place in the tropics, where the number of species on the animal and plant sides is high and forms entangled patterns of relationships difficult to unravel (Dáttilo & Rico‐Gray, 2018), much of what we know networks comes from research done in the simpler networks of the temperate zone (e.g., Bascompte, Jordano, Melián, & Olesen, 2003; Dáttilo & Rico‐Gray, 2018).

As expected, many aspects of the structure of species‐rich and species‐poor networks resemble one another (Dehling, 2018; Plein et al., 2013). Species richness aside, our investigation is motivated by a different issue—how are Neotropical frugivory networks structured in places significantly altered by human activity? Many investigations on the role of anthropogenic factors have examined the detrimental effects of human activities on different elements of biodiversity (Figueroa et al., 2020; Moreira, Ferreira, Lopes, Gomes, & Boscolo, 2018), and the role of habitat loss and fragmentation in frugivory networks (Gonzalez & Loiselle, 2016; Memmott, Waser, & Price, 2004; Ramos‐Robles, Andresen, & Díaz‐Castelazo, 2018). Nonetheless, our understanding of frugivory networks in these anthropogenically simplified ecosystems is comparatively less of what we know about the ones in a more pristine condition. These ecosystems include a wide variety of habitats that range from the moderately disturbed to the actively managed such as degraded forests (Ramos‐Robles et al., 2018), forest–farmland edges (Menke, Böhning‐Gaese, & Schleuning, 2012), agricultural fields and orchards (Plein et al., 2013), and city parks (Costa Cruz, Albino Ramos, da Silva, Tenreiro, & Huttel Heleno, 2013).

We chose a Neotropical, periurban park (a “simplified ecosystem”) to conduct our research. Simplified ecosystems, according to Western (2001), Fortuna & Bascompte (2006), and Figueroa et al. (2020), share a common set of traits that include simple food webs, homogenous landscapes, and require high nutrient and energy inputs in order to be maintained. Some elements, in turn, are gained, such as exotic species (Costa Cruz et al., 2013). In spite of its accessibility as study sites, we found very few examples of how frugivory networks are structured in urban (embedded within a city) and periurban (located in the fringes of a city) parks in the Neotropics (e.g., MacGregor‐Fors & Escobar‐Ibáñez, 2017; Oliveira, Franchin, & Junior, 2015).

We hypothesized that one such park would have a frugivory network of small size, composed with a few tens of species given its periurban nature. As a whole, however, we expected that network‐level features would reflect some of the general structural patterns of more complete networks (e.g., of those found in “natural” habitats) but would differ from them in some fundamental aspects. We also expected that generalists, exotic, managed, and abundant species would play the central roles in this frugivory network.

The aim of this paper is to assess which of the properties found on fully functional and disturbed frugivory networks are present in a simplified ecosystem. Our deconstructing logic envisions identifying network properties that prevail when some elements are missing. In this paper, we (a) characterize the frugivory network between birds and plants of a Neotropical periurban park, (b) compare standard network‐ and species‐level metrics to those known from functional and disturbed networks elsewhere, and (c) identify traits that persist or get lost in this network given its condition as a simplified ecosystem.

## 2 METHODS

### 2.1 Study site

**FIGURE 1:**
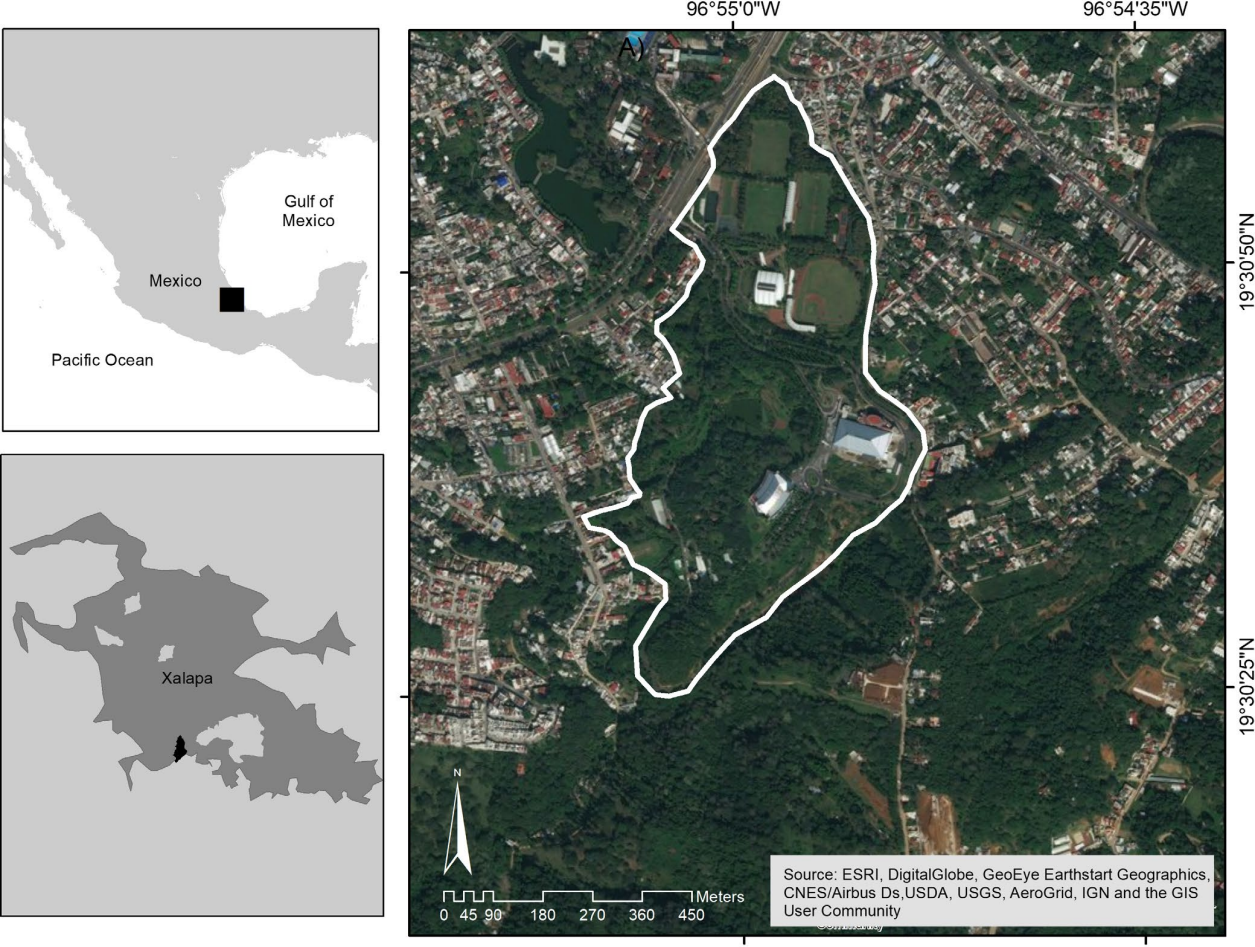
An anthropogenically simplified ecosystem, the Universidad Veracruzana's Campus para la Cultura, las Artes y el Deporte in Xalapa, Veracruz, Mexico. This Neotropical periurban park is located at the interface between the ca. 600,000 people city and a large matrix of coffee fincas and second growth vegetation that surrounds it. Both, this park and the adjacent habitat patches, are part of an archipelago of protected areas that abuts the city. Figure courtesy of Sara P. Ibarra‐Zavaleta, Universidad Veracruzana

We studied the frugivory network of the Universidad Veracruzana's Campus para la Cultura, las Artes y el Deporte (hereon UV‐CCAD). The UV‐CCAD is located in the outskirts of the City of Xalapa, Veracruz, Mexico, between 19°30′25″–19°31′11″N and 96°55′11″–96°54′48″W, at an average elevation of 1,417 masl. We consider this Neotropical, periurban park an anthropogenically simplified ecosystem because of its small size (33‐ha), relative position within the city, recent vegetation history, and active management (Figure 1).

The history of this periurban park dates back two decades. The UV‐CCAD was established in 1997 in an area formerly occupied with soccer fields (at the time called Campos Juárez) and the pasture fields of the Veracruz Mounted Police, a state regiment that kept horses, mules, and cows for decades before its acquisition by the Universidad Veracruzana.

Twenty years ago, its compacted clay soils hosted mainly forage grasses for cattle and scattered trees of huizache (*Acacia pennatula*) whose seeds are dispersed by cows and left to grow by cattle managers to provide shade for their herd. It was until the founding of the first facility, a central library, 1 year later, that the greenspaces of this campus received some management. Roughly one‐half of the area was left to continue its process of natural secondary succession and the other half was converted into sports facilities, several buildings, artificial impoundments, ornamental lakes, and managed gardens with native and exotic ornamental species.

The original vegetation, probably well over 100 years ago, was tropical montane cloud forest (Castillo‐Campos, 1991). This park has very few arboreal elements of its original vegetation, including a handful of old oaks (*Quercus* spp.), sweetgums (*Liquidambar macrophylla*), sycamores (*Platanus mexicana*), marangolas (*Clethra mexicana*), and other cloud forest trees. The current condition of natural growth areas, however, is of a well‐developed secondary vegetation (locally termed “acahual”) with a canopy of about 12–15 m, younger age clearings, interspersed coffee, citrus, banana, and other species of economic interest, a growing number of patches with advanced second growth vegetation, and a few elements of mature cloud forest.

The UV‐CCAD is spatially connected to a mosaic of second growth vegetation of >500 ha, composed primarily of shade‐grown coffee and second growth vegetation (Figure 1). Although the UV‐CCAD is now part of an archipelago of protected areas that surround the city of Xalapa (Gaceta Oficial del Estado, 2015), the layer of legal protection of these lands may not suffice to maintain its current condition given the fact that the protected area was declared atop private properties and this means the park periphery will very likely be engulfed by urban growth within the next 15–20 years.

### 2.2 Interaction records

Because the fruiting phenology of plants in our study site is unknown, our fieldwork took place during three sampling periods of equal duration and separated from each other by 2 months over the course of 1 year (three periods of 2 months on, 2 months off). The three periods of study were December 2016–January 2017, April–May 2017, and August–September 2017.

We collected focal observations of birds throughout the UV‐CCAD. These were collected following all walkable trails and roads. Our focals consisted in records obtained with 8 or 10 × binoculars in the early hours of the day (mostly from about 07:00–10:00), following individual birds or flocks of them as they foraged in the park's vegetation for a total of 31 days of focal observations (ca. 90 hr of total field effort) evenly distributed among the three sampling periods. During each daily period of observation, the linear distance walked was ≈1 km, and we estimate we sampled 3–21 fruit‐bearing plants of different forms (shrubs, trees, palms, vines, epiphytes, and hemiparasites) per survey session. Five independent observers were involved all of the focal observations.

For the purpose of this study, a frugivory interaction was an event involving one bird feeding on one fruit. We made all efforts possible to avoid sampling the same individual birds and walked away from possible pseudoreplicates (our field observations contain relatively few records of the same species on a given sampling session). We made no assumptions as to which species were true frugivores, as from our previous experience we found that many birds that are assumed to be “pure” insectivores actually feed on fruit. Once a bird was found feeding on fruit, we recorded the interaction in a datasheet and either identified the tree or shrub (if known) or collected a specimen for later identification.

We made a small reference collection of botanical material by pressing and drying specimens obtained during bird focal observations and by walking the area of the entire UV‐CCAD in search for known or suspected plants with an ornithocorous fruit syndrome. From plant material, we made a specimen, fruit, and seed reference catalogue, and also took photographs of fresh material for future use. The basic product of our focal observation work was an adjacency matrix (*A*) that summarizes the interactions recorded by placing each plant species (*j*) in one column, a bird species in each row (*i*), and the number of recorded interactions in the intersecting cell (*A_ij_*). No‐records were filled with zeroes (Bascompte et al., 2003; Falcão, Dáttilo, & Rico‐Gray, 2016).

### 2.3 Network completeness

During the collection of focal bird data and plant collections for our catalogue, we found that the number of species participating in this frugivory network is quite limited, possibly including only a few tens of species in either interacting group. The perception of this network as species‐poor comes from our familiarity with this park's surrounding habitats and the documented potential pool of bird and fruit‐bearing plant species documented therein (e.g., González‐García, Straub, Lobato García, and MacGregor‐Fors (2014), González‐García, Straub, Lobato García, MacGregor‐Fors, and Santiago‐Alarcón (2016) list 340 species of birds for the city of Xalapa, whereas Castillo‐Campos (1991) documents the flora of the municipality of Xalapa as well over 1,000 spp.).

Because we are faced with a simplified ecosystem, assessing the completeness of our network sampling became a critical first step required to precede analyses with a robust method to handle a small universe of interacting species (Casas, Bastazini, Debastiani, & Pillar, 2016; Chacoff et al., 2012; Hyde, Stewart, & Miller, 2014). We estimated accumulation curves via a R package for Hill numbers (an estimate of the effective number of species as a function of sampling effort) called iNEXT (Hsieh, Ma, Chao, & McInerny, 2016). This package can generate seamless rarefied (interpolated) species accumulation curves based on observed data and is also able to project a rarefied extrapolation of the predicted number of species or species interactions given a larger sampling effort. Those calculations are accompanied by 95% confidence intervals.

We applied the method of Hsieh et al. (2016) to both, the bird and the plant groups, as well as to the observed and predicted interactions between them. Hsieh et al. (2016) validate and discuss in great detail three widely used elements of the Hill number family, such as species richness, Shannon, and Simpson diversity indices, and highlight the benefits of its use when compared to other widely used methods to estimate species richness such as those of Colwell, Chang, and Chang (2004) and his software project EstimateS. The code of Hsieh et al. (2016) has a function to extrapolate its values given a hypothetical sample with two or three times as many focal records, enabling its users to estimate a predicted number of species and whether its actual (interpolated) or simulated (extrapolated) curve reaches an asymptote.

### 2.4 Network structure and metrics

The scope of most recent analyses of frugivory networks involves building bipartite networks from adjacency (or one‐mode) tables into two‐mode networks. One column (also known as level or group) typically depicts plants and the other the interacting animal group (pollinators, herbivores, frugivores, etc.), whereas links between them represent the number of observed interactions (Antoniazzi, Dáttilo, & Rico‐Gray, 2018; Bascompte & Jordano, 2014).

We built an interaction matrix to characterize the bipartite network and made calculations of several metrics using the R package BIPARTITE (Dormann et al., 2008; Dormann et al., 2009). These metrics provide values of two types of descriptors: network‐ and species‐level. The BIPARTITE output includes a comparison of the observed value of network‐level indices with a null model (i.e., expected value) and generates a *p* value, akin to a classic statistic's significance test (Dormann et al., 2008).

### 2.5 Network‐level indices

The first and most simple metric is *Network size* (*S*), the sum of the number of plants (P) and birds (B) in the network (P + B). The product of both terms (P × B) yields an estimated number of possible links. A second informative metric is the mean number of *links per species* (${\bar{L}}_{x}$) that reflects the degree of specialization of species that belong to both groups.

A recent review of network metrics by Dehling (2018), highlights the value of two network‐level metrics over many other calculations generated using this analysis approach. These are nestedness and modularity. *Nestedness* is a core metric that reflects a well‐known feature of bipartite networks: the fact that specialists interact closely with subgroups of generalist species (nestedness is referred to by its acronym NODF in many recent papers—NODF stands for Nested metric based on Overlap and Decreasing Fill, Almeida‐Neto, Guimarães, Guimara, Loyola, & Ulrich, 2008; Falcão et al., 2016). NODF is a measure of subordination of the less‐connected species to those who have a highly connected pattern. *Modularity* (*M*) is another essential function that quantifies how many groupings, compartments, or modules can be found in a network. Modules are subsets of species that interact with each other more that with those in the remaining modules and reflect phylogenetic and functional affinities among them (Olesen, Bascompte, Dupont, & Jordano, 2007). *M* is calculated with an algorithm developed by Dormann and Strauss (2014), in which a network with high values of modularity has interactions that fall primarily within modules and a few to none outside of it (Dormann, Fruend, & Gruber, 2020).

### 2.6 Species‐level indices

These indices are suited to describe the diversity and organization of species interactions in a network, and can be used to determine structural and functional roles of individual species (Dehling, 2018). *Degree* (also referred to in the literature as centrality, Bascompte & Jordano (2006)) is the number of links per species in the network. Species with a high degree distribution value are those interconnected at higher frequencies with species in the other group (Bascompte & Jordano, 2006; Freeman, 1977).

Last, *species strength* (*SS*) is the sum of individual species dependencies (d′) for the entire set of species in the other group (Dehling, 2018). Examples of this are species in one group that are highly connected to a set of species in the other group, forming a module. When a species has a high d′ value, it means a species is most likely a generalist of a high functional importance; hence, its removal from a network means that a key component of it has been lost and the network will likely have severe functional consequences (Ramos‐Robles et al., 2018; Rumeu et al., 2017; Vázquez et al., 2007).

High degree species, generalists that interact with a diverse set of species, may become *network hubs* when its pattern of interconnectedness spreads throughout the entire network, whereas those highly connected within the same subset of species are considered *module hubs* (Mello et al., 2011). Two scores are used to determine these roles: *z*‐scores measure the degree of connection of species within a module, and *c*‐scores quantifies how even those connections are across all modules (Dáttilo et al., 2016). *Z‐* and *c*‐scores have the threshold cutoff values to separate the role of species within and among the modules at the 95% percentile (based on the mean, from lowest to highest values) and enables analysts to classify species as peripherals (with a few interactions with other species), connectors (connects several modules to each other), module hubs (has several interactions within its module), or network hubs (the species is a connector and has several interactions in the module) (Dáttilo et al., 2016; Olesen et al., 2007).

## 3 RESULTS

### 3.1 Network completeness

**FIGURE 2:**
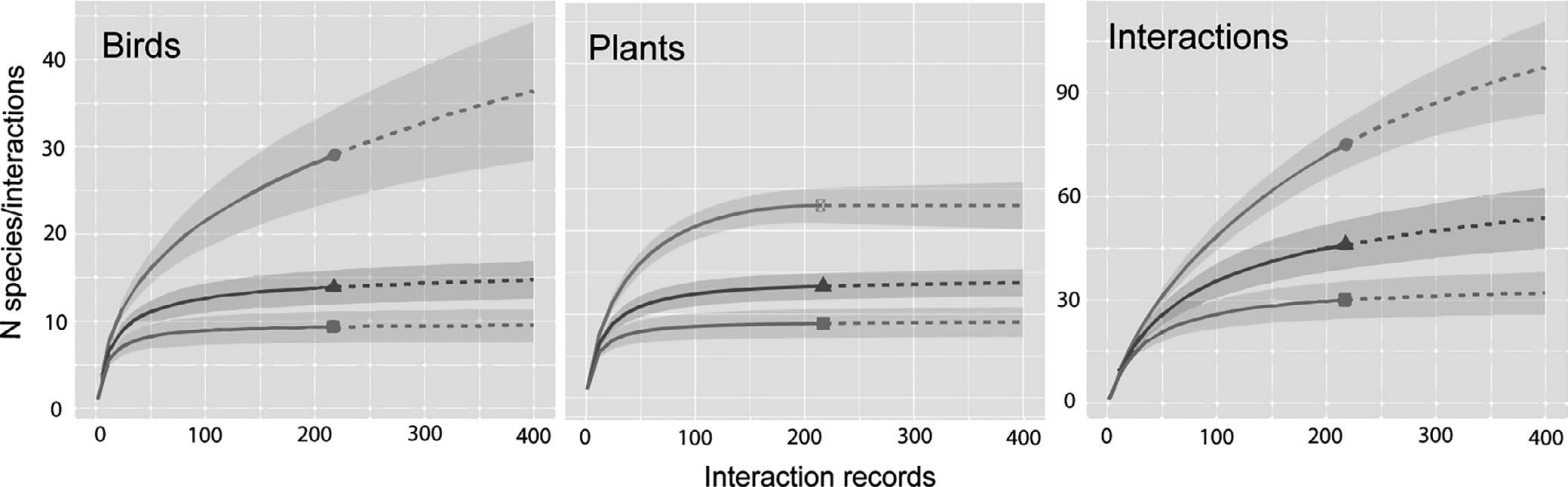
Bird and plant species richness, and frequency of species interactions, in the frugivory network of the UV‐CCAD Neotropical periurban park in Xalapa, Veracruz, Mexico. The solid line is the interpolated observed frequency; the dotted line, an extrapolation given twice the sampling effort. Circles = species richness or number of interactions, triangles = abundant species or abundant interactions, squares = dominant species or dominant interactions. Gray shading around interpolated and extrapolated lines (solid and dotted, respectively) represent 95% confidence intervals. See text for a detailed description of the calculation of these accumulation curves

We recorded 217 interactions between birds and plants. The frugivory network is composed of 29 species of birds that consumed 23 species of plants (network size *S* = 52 species, Figure 2). Our sampling effort yielded a fairly saturated network, with differences between groups. The accumulation curves for birds, plants, and interactions show varying degrees of sampling saturation. For birds, the observed number of species (*n* = 29) is higher than that of plants. When simulating the number of focals to 400, species richness can increase to a projected total of 36 (a 24% increase). Abundant and dominant species, however, are well represented in our sampling, showing the clear reaching of an asymptote in both cases. Among plants, the story is different. Our observed species accumulation curve reaches an asymptote at 23 species. Simulating the collection of 400 interaction records does not increase at all the number of species in this network. The least complete accumulation curve, as expected, is that of interactions, with 75 unique links. When extrapolating the curve to 400 focal observations, the resulting number of interactions is 97, a 29% increase over the number recorded.

### 3.2 Plant and bird species in this network

In the plant group, three species account for 51% of the total number of interactions. These are an exotic, managed palm (*Phoenix canariensis*), a hemiparasite mistletoe (*Phoradendron* sp.), and a nightshade (*Witheringia stramoniifolia*). Plants recorded belong to 17 families. Nearly half of them are shrubs and trees characteristic of early and mid‐successional cloud forest flora (Table S1).

Among birds, four species stand out as dominant (55% of all interactions): social flycatcher (*Myiozetetes similis*), clay‐colored thrush (*Turdus grayi*), common chlorospingus (*Chlorospingus flavopectus*), and a single seasonal visitor, the gray catbird (*Dumetella carolinensis*) (birds in black boxes in Figure 2). Birds recorded belong to 12 families. Although most of the birds are year‐round residents (72%), Neotropical migrants (present only from about September through April) account for a sizable proportion (28%) of the species and interactions of this network (Table S2).

### 3.3 Metrics of network structure

Our bird–plant network is not nested (Table 1). Species are aggregated in eight clearly differentiated modules, varying in the number of species represented in each of them. For both, plants and birds, modules range from 1–8 species per module, although their mean number of species per module value varies from a ${\bar{x}}_{\text{plants}}$ = 2.87 to ${\bar{x}}_{\text{birds}}$ = 3.62. Two modules contain all central species. Relatively few interactions fall outside these groupings (Figure 4).

**TABLE 1:** Network‐ and species‐level indices of the frugivory network between birds and plants of a simplified ecosystem, a Neotropical, periurban park in Xalapa, Veracruz, Mexico

Metric		Value	
Network‐level	Observed	Null model	*p*
Nestedness (NODF)	20.65	45.84	<0.001
Modularity (*M*)	0.54	0.25	<0.001
Species‐level	$\bar{x}$	*σ*	Range
Degree			
Plants	9.43	12.13	2–51
Birds	7.48	11.06	1–49
Species strength (*SS*)			
Plants	1.25	1.22	0.05–4.36
Birds	0.79	1.16	0.02–4.37

### 3.4 Species‐level descriptors

The mean number of links of species per species is low, ${\bar{L}}_{x}$ = 1.4. The network is asymmetric, with a lower mean number of links for birds than for plants (Table 1).

Four species of birds and a single species of plant play key module roles in this network. Among birds, a single species (*Turdus grayi*) is generalist enough to play the module role of network hub. Three more species, *Euphonia hirundinacea*, *Myiozetetes similis* (both resident), and *Dumetella carolinensis* (a migrant), have high *z*‐score values that place them as module hubs (Figure 5). The role resident and migrant species play in this network, however, does not seem to differ (Figure S3). On the plant side of the network, a single species acts as module hub, *Phoenix canariensis* (Figure 5), with no apparent differences in the module roles between natives and exotics (Figure S4). Species strength (*SS*) is also very dissimilar between birds and plants, much higher in the latter (Table 1).

## 4 DISCUSSION

This frugivory network contains a low number of species and interactions given its condition as a simplified ecosystem. In this study, however, we found that one of the network‐level properties (modularity) maintains values similar to those of well‐conserved networks, whereas the other (nestedness) is not. Species‐level properties examined here (richness, degree, and species strength) exhibit low values.

### 4.1 This network is poor in species and interactions

It could be argued that a Neotropical frugivory network with the size we report here (*S* = 52 species) and its structural properties could be an artifact of incomplete sampling instead of a species‐ and interaction‐poor system (e.g., Falcão et al., 2016). When comparing our species accumulation curves to those of other studies, we find some resemblance in the degree of saturation of them. García‐Robledo, Erickson, Staines, Erwin, and Kress (2013), for example, found an interaction pattern that is similar to our work: species accumulation curves are saturated in plants, below reaching an asymptote for animals, and well below saturation for interactions. In both cases, we consider abundant and dominant species as well recorded.

While plant and bird species richness curves have a simple explanation because of its relationship with the overall reduced ecosystem richness of our study site, the latter issue, the saturation of interactions, is less clear, as it has not received sufficient attention in the literature. In theory, the expected number of interactions is the product of P × B. This simplistic view of the potential number of interactions misses considering a number of forbidden interactions (those that are not possible due to temporal, spatial, morphological, or other constraints; Bascompte & Jordano, 2014; Olesen & Jordano, 2002; Vázquez, Blüthgen, Cagnolo, & Chacoff, 2009; Vizentin‐Bugoni et al., 2018). This means that many interactions in our network are impossible and that this curve overestimates the number of interactions to an unknown degree. Until this issue is addressed, we will continue obtaining artificially low connectance (*C*) values in these networks (e.g., the connectance we found in this study, *C* = 0.11, is contained within the range of 0.07–0.27 reported by de Almeida & Mikich, 2017). In our study, abundant and dominant species at the core of this network, however, are sufficiently sampled (Figure 2).

### 4.2 Generalists and exotics dominate this plant‐bird network

At the core (e.g., species with high values of d′, *c*‐ and *z*‐scores) of this frugivory network lie three species of plants that represent three groups that thrive under anthropogenically simplified situations (e.g., López de Buen & Ornelas, 2001). These include the cultivar of an exotic palm popularly used for landscaping (Barrow, 1998), a bird‐dispersed hemiparasite (Hernández‐Ladrón de Guevara, Rojas‐Soto, López‐Barrera, Puebla‐Olivares, & Díaz‐Castelazo, 2012), and a widespread pioneer native nightshade (Sousa‐Pena, 2001, Figure 3). Among birds, the core of interacting species is formed by three species that are widespread generalists and a fourth one (*Chlorospingus flavopectus*) that is known as a mid‐successional common bird of cloud forests that often leads multispecies flocks (Stotz, Fitzpatrick, Parker, & Moskovits, 1996, Figure 3).

**FIGURE 3:**
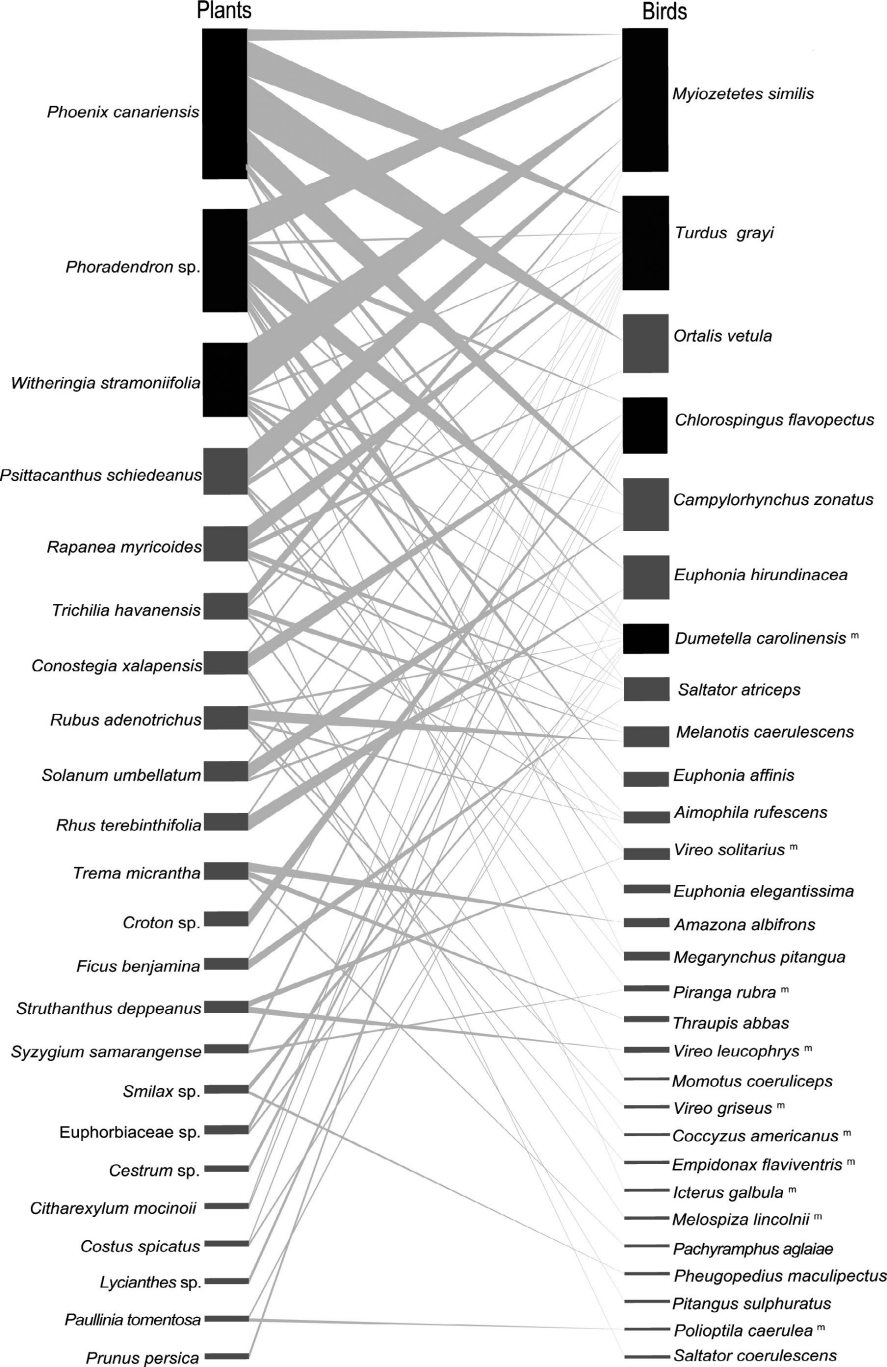
The bipartite frugivory network between 23 species of plants and 29 species of birds of the UV‐CCAD Neotropical periurban park in Xalapa, Veracruz, Mexico. Size of species boxes represents its frequency in observed interactions; the thickness of connecting lines denotes the frequency of repeated interactions or links. Boxes in black are of those of nuclear species in both groups. Superindex^M^ identifies Neotropical migratory bird species

In the analysis of mechanisms and functional consequences of frugivory, the traits exhibited by generalists and exotics (such as morphological and behavioral) have been placed at the lower end of a spectrum that leads to simplification and ecosystem decay (e.g., Lebrija‐Trejos, Pérez‐García, Meave, Bongers, & Poorter, 2010). Managed exotic species, in particular, have been identified as elements that increase generalization and contribute to simplify functional traits of ecosystems (García, Martínez, Stouffer, & Tylianakis, 2014), whereas increases in specialists, and the correspondent decreases in generalists, are viewed as gains in ecosystem resilience (Bastazini, Debastiani, Azambuja, Guimarães, & Pillar, 2017). Because this periurban park has actually increased its vegetation cover and diversity of ecological interactions in the most recent 20 years, our observations fit with the generalist‐dominated pattern of early secondary succession (e.g., Lebrija‐Trejos et al., 2010).

### 4.3 Network structure metrics send a mixed signal of stability; species metrics reveal unclear functional roles

The architecture of frugivory networks is expected to be more robust with increased complexity (Bascompte et al., 2003; Jordano, 1987). Previous studies analyzing the structure of herbivory, pollination, plant protection, and frugivory have described network‐level patterns that coincide with some of the features we found in our work (e.g., Dáttilo et al., 2016; Dáttilo & Rico‐Gray, 2018; McQuaid & Britton, 2013; Zanata et al., 2017). On the other hand, our species‐level characteristics are similar to those of anthropogenically altered systems (e.g., Costa Cruz et al., 2013; Plein et al., 2013).

In a particularly illuminating recent meta‐analysis of frugivory networks throughout the Neotropics, de Almeida and Mikich (2017) analyzed 17 independent datasets with the aim of finding generalized patterns of network structure. De Almeida and Mikich (2017) assembled networks of different sizes (*S* values ranging from 64–415 species) and calculated metrics of its main descriptors using a very similar topology and methods of our paper. When compared to the work of de Almeida and Mikich (2017) our network‐level descriptors fall within the known ranges that characterize a network that is deterministically (nonrandomly) assembled in spite of being poor in species (Solé & Montoya, 2001).

Networkwise, two descriptors are worth highlighting because of its intrinsic importance in defining mutualistic networks (Dehling, 2018). Nestedness is a common feature of nonrandom networks (Bascompte et al., 2003; Krishna, Guimarães, Jordano, & Bascompte, 2008). Different meta‐analyses report significant nestedness in the majority of the networks studied, from 58% of the networks with statistically significant nestedness (using the NODF index) in the paper of de Almeida and Mikich (2017), to 75% of the networks summarized in Bascompte et al. (2003). Although our NODF values fall mid‐range within the known published values (de Almeida & Mikich, 2017), our observed value is lower than the null model, which means that our network has not developed the degree of disturbance resilience of better‐preserved habitats (Bascompte et al., 2003; Plein et al., 2013). Moreover, Tylianakis and Morris (2017) consider nestedness as a metric related to specialists, a characteristic lacking in this network.

**FIGURE 4:**
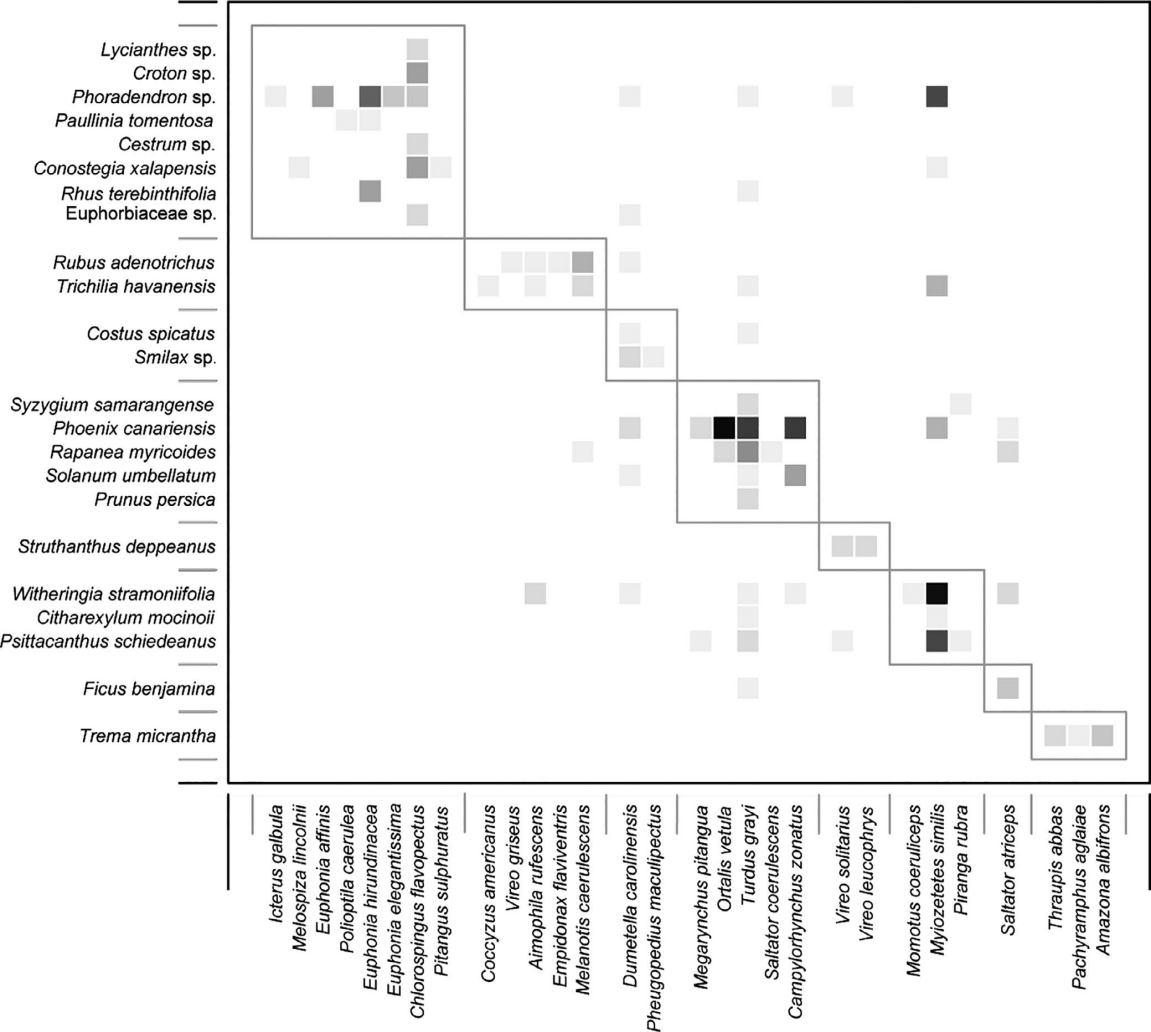
Modularity of bird and plant species interactions at the UV‐CCAD, a Neotropical periurban park. The modules are generated using the algorithm QuaBiMo (Dormann & Strauss, 2014; García et al. 2014). Each square is a link between species, with the darkest colors representing a higher frequency of interactions. Dark rectangles frame modules

Modularity is the second structural descriptor that is diagnostic of functional networks. Modularity, a form of compartmentalization, is hypothesized to be a result of temporal, morphological, or phylogenetic drivers that aggregate species in semidiscrete groupings (Lewinson, Prado, Jordano, Bascompte, & Olesen, 2006). Modularity is a descriptor that may also be ubiquitous in other types of networks. Olesen et al. (2007), for example, found significant *M* values in 57% of the pollination networks (*n* = 51 networks). Our network is highly modular (Figure 4, Table 1), approaching the upper‐third of the known value range reported by de Almeida and Mikich (2017), which also means the relative contribution of generalists can be seen in this metric. Our work, and the meta‐analyses of Olesen et al. (2007) and Almeida and Mikich (2017), determined modularity using binary networks. We are aware that other approaches, such as that of Schleuning et al. (2014) using a different algorithm to generate a weighted modularity, are capable of associating species traits such as seasonality and phylogeny to this network metric.

**FIGURE 5:**
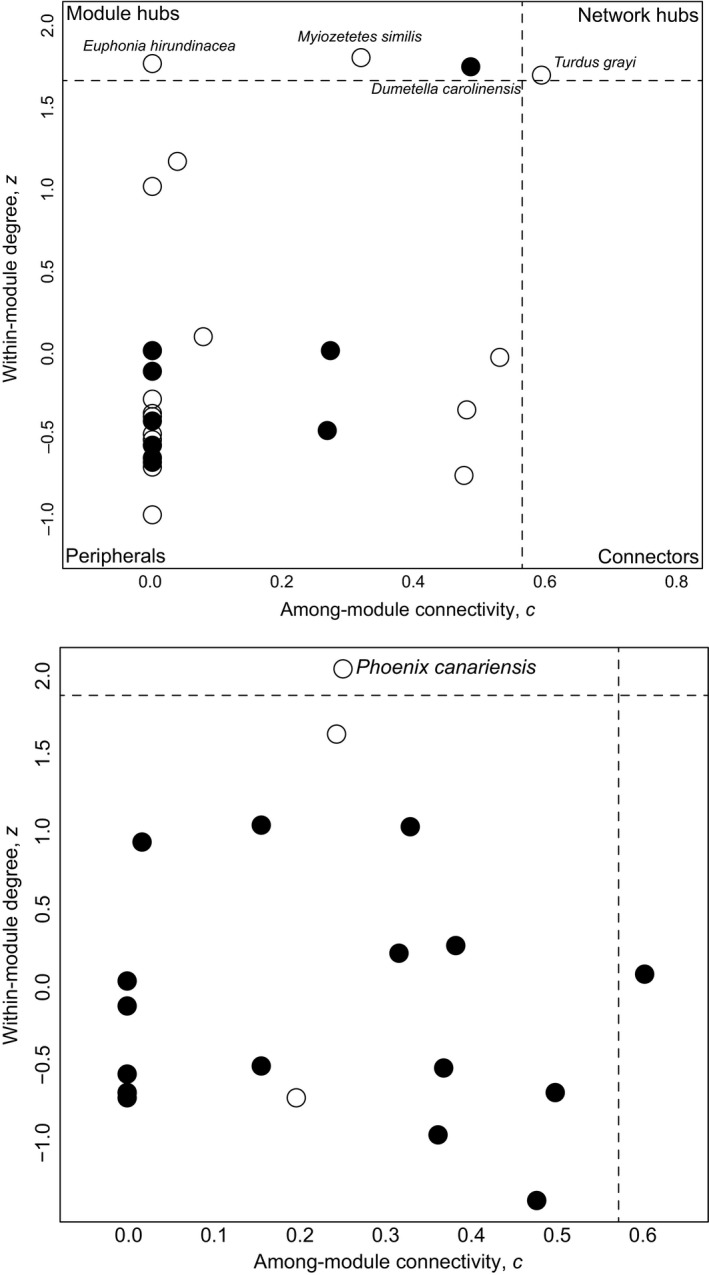
Module roles of birds (top) and plants (bottom) in the frugivory network of a Neotropical, periurban park. *Z*‐values quantify the number of interactions, whereas *c*‐values provide a value for among‐module connectivity. White circles = resident species; black circles = migratory birds. For plants, black circles correspond to native plants, white circles = exotic/managed plants

Species‐level metrics, in turn, reveal generalists with unclear functional roles. For example, the plant and bird groups in this network are not reciprocally specialized, which is a pattern found in many other works (e.g., Dehling, 2018). The roles of both groups (Figure 5) reveal very few species act as module or network hubs, and the overwhelming majority of them (85% of the birds and 95% of the plants) are peripherals. Degree distribution values show plants have a higher mean number of links per species and are more tightly interconnected. *SS* values are very low in both groups (Table 1), a metric where the range of values goes from 0–5, where a value of 5 is for stronger species. This value show plants have a comparatively higher network functional importance than birds (e.g., Sebastián‐González, 2017). Further comparisons with anthropogenically altered systems are difficult due to differences in data collection methods, the absence of a quantitative method to make comparisons among them, as well as differences in network and species metrics in use (e.g., Costa Cruz et al., 2013; Menke et al., 2012; Plein et al., 2013; Sebastián‐González, 2017).

In summary, our study shows that the two network‐level descriptors of our frugivory network fall between those of fully functional (significantly modularity) and anthropogenically altered (nonsignificantly nested) networks, whereas species‐level index values exhibit a random pattern. Modularity, hence, seems to be the most pervasive network metric in this anthropogenically simplified ecosystem undergoing secondary succession (Olesen et al., 2007; Plein et al., 2013).

Although urban and periurban parks are often viewed as ecologically incomplete systems (Costa Cruz et al., 2013; MacGregor‐Fors & Escobar‐Ibáñez, 2017), our results suggest some elements of ecological function are maintained or, in our case, acquired as succession progresses (Pellissier et al., 2018; Yang et al., 2018; Sebastián‐González, 2017). Managementwise, this park epitomizes the tension between esthetic interests (a view that tends to generalize and simplify species and interactions) and the process of natural succession that builds specialization and resilience from a less structured foundation of generalists. The study of simplified ecosystems allows researchers to distill some elements of its structure and function to its most basic elements and put to test the persistence and loss of its measurable properties.

## CONFLICT OF INTEREST

None declared.

## AUTHOR CONTRIBUTIONS

**Gabriela I. Salazar‐Rivera:** Conceptualization (equal); data curation (lead); formal analysis (equal); funding acquisition (supporting); investigation (equal); methodology (equal); project administration (supporting); resources (equal); software (lead); supervision (supporting); validation (supporting); visualization (lead); writing–original draft (supporting); writing–review and editing (supporting). **Wesley Dáttilo:** Conceptualization (equal); data curation (supporting); formal analysis (equal); funding acquisition (supporting); investigation (equal); methodology (equal); project administration (supporting); resources (equal); software (equal); supervision (equal); validation (equal); visualization (supporting); writing–original draft (supporting); writing–review and editing (supporting). **Gonzalo Castillo‐Campos:** Conceptualization (supporting); data curation (supporting); formal analysis (supporting); funding acquisition (supporting); investigation (equal); methodology (supporting); project administration (supporting); resources (supporting); software (supporting); supervision (equal); validation (equal); visualization (supporting); writing‐original draft (supporting); writing–review and editing (supporting). **Norma Flores‐Estévez:** Conceptualization (supporting); data curation (supporting); formal analysis (supporting); funding acquisition (supporting); investigation (supporting); methodology (supporting); project administration (supporting); resources (supporting); software (supporting); supervision (equal); validation (supporting); visualization (supporting); writing–original draft (supporting); writing–review and editing (supporting). **Brenda Ramírez García:** Conceptualization (supporting); data curation (supporting); formal analysis (supporting); funding acquisition (supporting); investigation (supporting); methodology (supporting); project administration (supporting); resources (supporting); software (supporting); supervision (supporting); validation (supporting); visualization (supporting); writing–original draft (supporting); writing–review and editing (supporting). **Ernesto Ruelas Inzunza:** Conceptualization (lead); data curation (equal); formal analysis (equal); funding acquisition (equal); investigation (equal); methodology (lead); project administration (equal); resources (equal); software (supporting); supervision (lead); validation (lead); visualization (equal); writing–original draft (lead); writing–review and editing (lead).

## Supporting information

Table S1

Table S2

Figure S1

Figure S2

## Data Availability

The complete dataset of this research, including its metadata, is located in the data repository Dryad (https://doi.org/10.5061/dryad.12jm63xv6).
