# Supplementary material for: The frugivory network properties of a simplified ecosystem: Birds and plants in a Neotropical periurban park
Source: Ecol Evol. 2020 Aug 4;10(16):8579–91. doi: 10.1002/ece3.6481 (PMC7452784; doi:10.1002/ece3.6481)
Supplement: Supplementary file 1 — Table S1 [file ECE3-10-8579-s001.docx]

**Table S1**

| Bird species | Family | Seasonal status | Interaction frequencies | *NODF* | *Ci* | *Zi* | *d´* |
| --- | --- | --- | --- | --- | --- | --- | --- |
| *Myiozetetes similis* | Tyrannidae | R | 49 | *0.72* | *0.32* | *1.79* | *0.44* |
| *Turdus grayi* | Turdidae | R | 32 | *2.18* | *0.59* | *1.68* | *0.28* |
| *Ortalis vetula* | Cracidae | R | 20 | *-0.24* | *0.00* | *-0.29* | *0.49* |
| *Chlorospingus flavopectus* | Passerellidae | R | 19 | *0.54* | *0.04* | *1.15* | *0.75* |
| *Campylorhynchus zonatus* | Troglodytidae | R | 18 | *0.38* | *0.08* | *0.08* | *0.46* |
| *Euphonia hirundinacea* | Fringillidae | R | 15 | *-1.03* | *0.00* | *1.75* | *0.62* |
| *Dumetella carolinensis* | Mimidae | M | 10 | *0.98* | *0.48* | *1.73* | *0.26* |
| *Saltator atriceps* | Thraupidae | R | 8 | *-0.97* | *0.42* | *NA* | *0.41* |
| *Melanotis caerulescens* | Mimidae | R | 7 | *-0.11* | *0.53* | *-0.04* | *0.60* |
| *Euphonia affinis* | Fringillidae | R | 5 | *0.54* | *0.00* | *-0.40* | *0.39* |
| *Aimophila rufescens* | Passerellidae | R | 4 | *-0.10* | *0.48* | *-0.36* | *0.31* |
| *Vireo solitarius* | Vireonidae | M | 4 | *0.46* | *0.27* | *0.00* | *0.43* |
| *Amazona albifrons* | Fringillidae | R | 3 | *-1.12* | *0.00* | *1.00* | *0.81* |
| *Euphonia elegantissima* | Psittacidae | R | 3 | *0.51* | *0.00* | *-0.54* | *0.31* |
| *Megarynchus pitangua* | Tyrannidae | R | 3 | *-0.57* | *0.47* | *-0.76* | *0.14* |
| *Piranga rubra* | Cardinalidae | M | 2 | *-2.18* | *0.27* | *-0.49* | *0.48* |
| *Tangara abbas* | Thraupidae | R | 2 | *-1.19* | *0.00* | *0.00* | *0.71* |
| *Vireo leucophrys* | Vireonidae | M | 2 | *-1.08* | *0.00* | *0.00* | *0.81* |
| *Icterus galbula* | Cuculidae | M | 1 | *0.50* | *0.00* | *-0.68* | *0.10* |
| *Momotus coeruliceps* | Icteridae | R | 1 | *0.41* | *0.00* | *-0.51* | *0.18* |
| *Coccyzus americanus* | Momotidae | M | 1 | *-0.42* | *0.00* | *-0.43* | *0.44* |
| *Pachyramphus aglaiae* | Passerelidae | R | 1 | *-1.24* | *0.00* | *-1.00* | *0.54* |
| *Saltator coerulescens* | Polioptilidae | R | 1 | *-0.40* | *0.00* | *-0.71* | *0.37* |
| *Vireo griseus* | Thraupidae | M | 1 | *-0.59* | *0.00* | *-0.65* | *0.47* |
| *Pheugopedius maculipectus* | Tityridae | R | 1 | *-1.89* | *0.00* | *-0.38* | *0.72* |
| *Pitangus sulphuratus* | Troglodytidae | R | 1 | *-0.76* | *0.00* | *-0.58* | *0.47* |
| *Melospiza lincolnii* | Tyrannidae | M | 1 | *-0.76* | *0.00* | *-0.58* | *0.47* |
| *Polioptila caerulea* | Tyrannidae | M | 1 | *-1.18* | *0.00* | *-0.13* | *0.82* |
| *Empidonax flaviventris* | Vireonidae | M | 1 | *-0.54* | *0.00* | *-0.65* | *0.47* |
