# Supplementary material for: The frugivory network properties of a simplified ecosystem: Birds and plants in a Neotropical periurban park
Source: Ecol Evol. 2020 Aug 4;10(16):8579–91. doi: 10.1002/ece3.6481 (PMC7452784; doi:10.1002/ece3.6481)
Supplement: Supplementary file 2 — Table S2 [file ECE3-10-8579-s002.docx]

**Table S2**

| Plant species | Family | Biological form | Origin | Interaction frequencies | NODF | *Ci* | *Zi* | *d’* |
| --- | --- | --- | --- | --- | --- | --- | --- | --- |
| *Phoenix canariensis* | Arecacea | P | Introduced | 51 | 0.97 | 0.24 | 1.63 | 0.50 |
| *Phoradendron* sp. | Santalaceae | H | Native | 35 | 3.00 | 0.25 | 2.05 | 0.43 |
| *Witheringia stramoniifolia** | Solanaceae | T | Native | 25 | 1.62 | 0.33 | 1.04 | 0.38 |
| *Psittacanthus schiedeanus* | Loranthaceae | H | Native | 16 | 0.12 | 0.60 | 0.09 | 0.34 |
| *Rapanea myricoides* | Myrsinaceae | T | Native | 12 | 0.23 | 0.38 | 0.27 | 0.39 |
| *Trichilia havanensis* | Meliaceae | T | Native | 9 | 0.65 | 0.31 | 0.22 | 0.32 |
| *Conostegia xalapensis* | Melastomataceae | S | Native | 8 | 0.71 | 0.01 | 0.94 | 0.55 |
| *Rubus adenotrichus* | Rosaceae | S | Native | 8 | 0.19 | 0.15 | 1.05 | 0.74 |
| *Solanum umbellatum* | Solanaceae | T | Native | 7 | 1.02 | 0.36 | -0.50 | 0.43 |
| *Rhus terebinthifolia* | Anacardiaceae | C | Native | 6 | -0.09 | 0.15 | -0.49 | 0.52 |
| *Trema micrantha* | Ulmaceae | T | Native | 6 | -1.21 | 0.00 | NA | 1.00 |
| *Croton* sp*.* | Euphorbiaceae | T | Native | 5 | -0.45 | 0.00 | -0.54 | 0.58 |
| *Struthanthus deppeanus* | Loranthaceae | E | Native | 4 | -0.82 | 0.00 | NA | 0.89 |
| *Ficus benjamina* | Moraceae | T | Introduced | 4 | 0.53 | 0.14 | NA | 0.51 |
| *Syzygium samarangense* | Myrtaceae | T | Introduced | 3 | -0.35 | 0.19 | -0.70 | 0.39 |
| Unidentified | Euphorbiaceae | S | Introduced | 3 | 0.54 | 0.49 | -0.66 | 0.34 |
| *Smilax* sp. | Smilacaceae | T | Native | 3 | -0.28 | 0.00 | -0.11 | 0.68 |
| *Lycianthes* sp*.* | Solanaceae | T | Native | 2 | -0.43 | 0.00 | -0.66 | 0.38 |
| *Paullinia tomentosa* | Paulowniaceae | T | Native | 2 | -1.15 | 0.00 | 0.04 | 0.63 |
| *Prunus persica* | Rosaceae | T | Native | 2 | 2.15 | 0.00 | -0.70 | 0.24 |
| *Cestrum* sp*.* | Solanaceae | T | Native | 2 | -0.48 | 0.00 | -0.66 | 0.38 |
| *Citharexylum mocinnoi* | Verbenaceae | T | Native | 2 | 0.76 | 0.47 | -1.36 | 0.00 |
| *Costus spicatus* | Costaceae | S | Native | 2 | 0.67 | 0.36 | -0.94 | 0.21 |
