# Supplementary figures and images for: The frugivory network properties of a simplified ecosystem: Birds and plants in a Neotropical periurban park
Source: Ecol Evol. 2020 Aug 4;10(16):8579–91. doi: 10.1002/ece3.6481 (PMC7452784; doi:10.1002/ece3.6481)

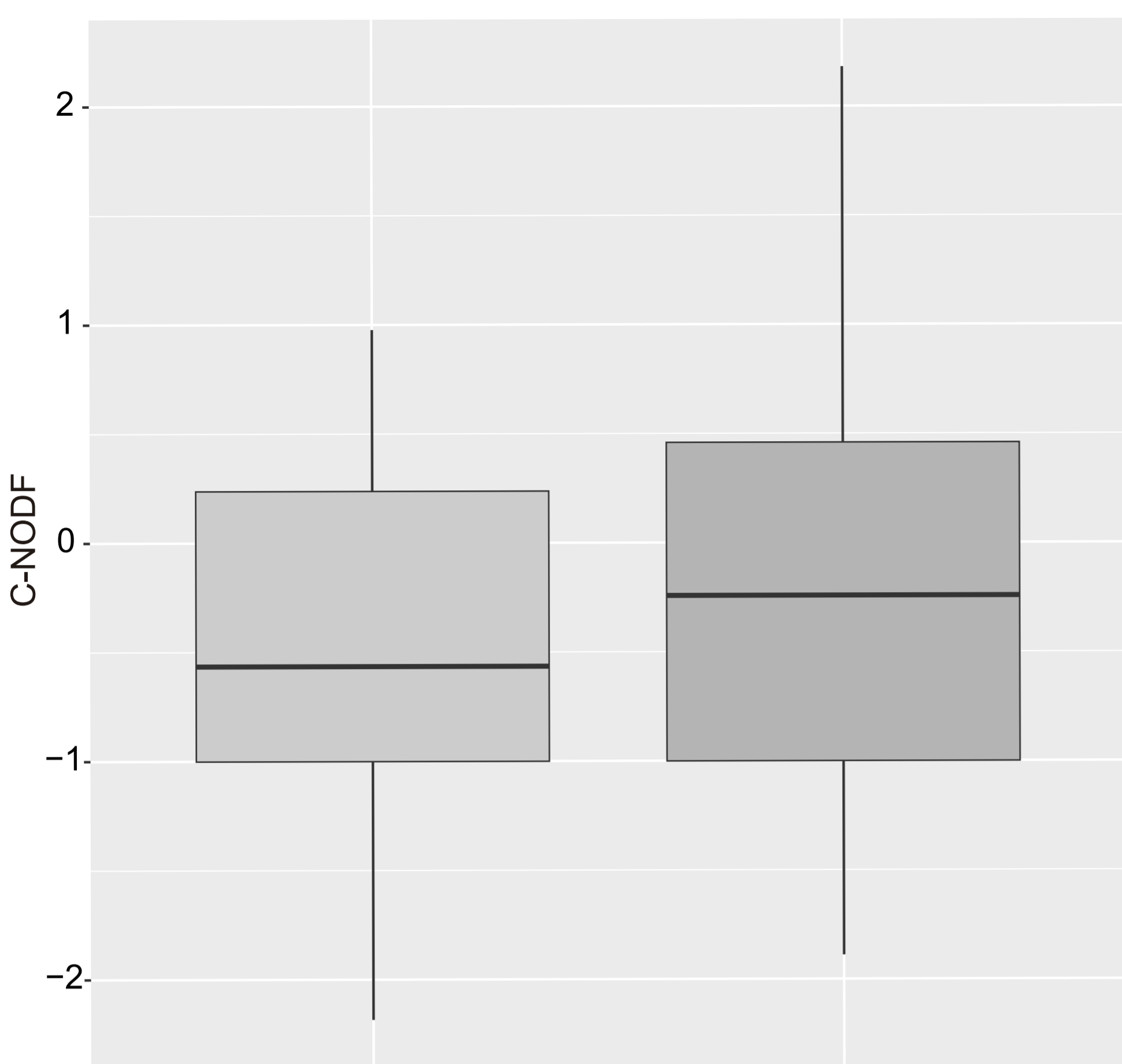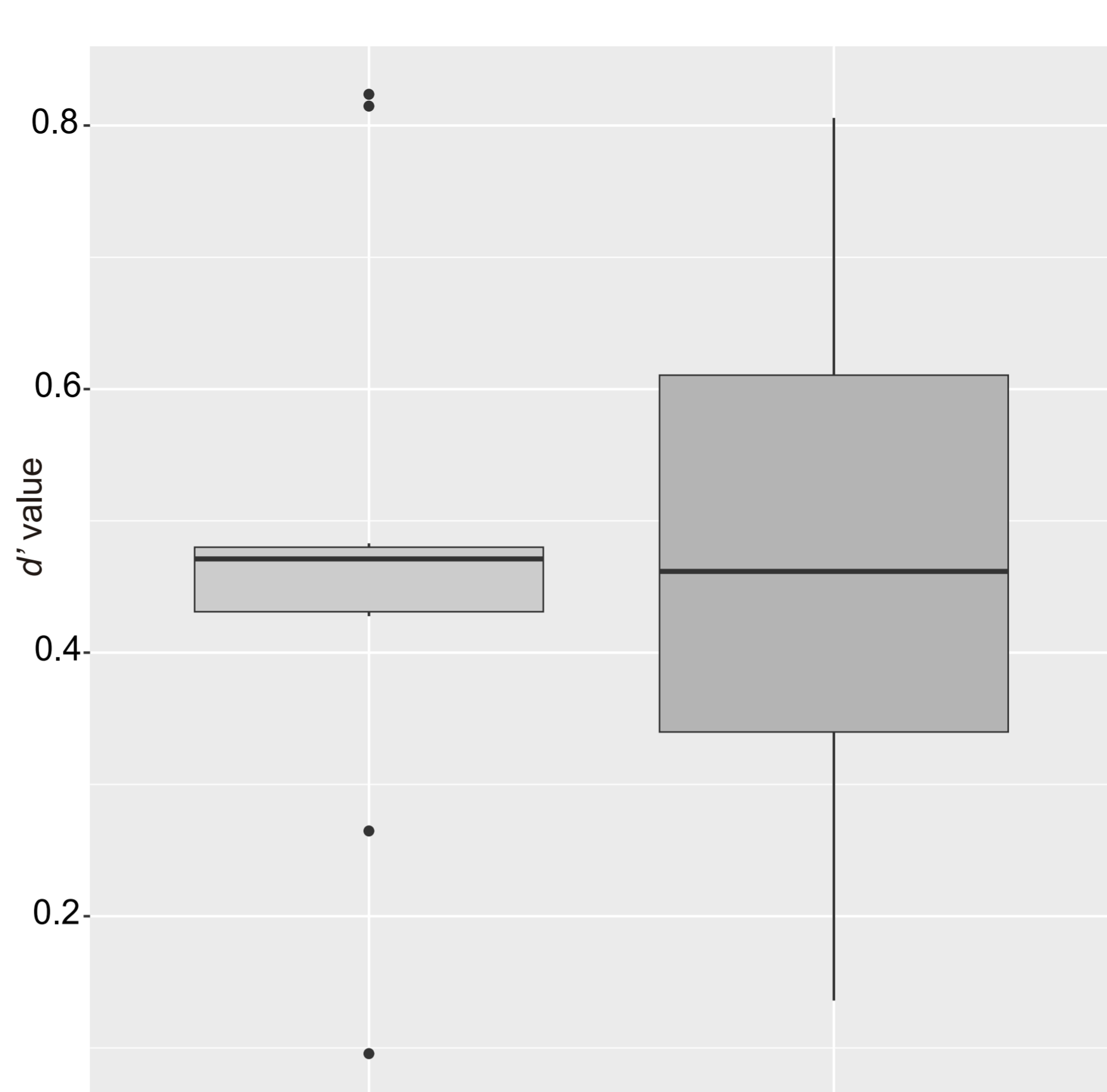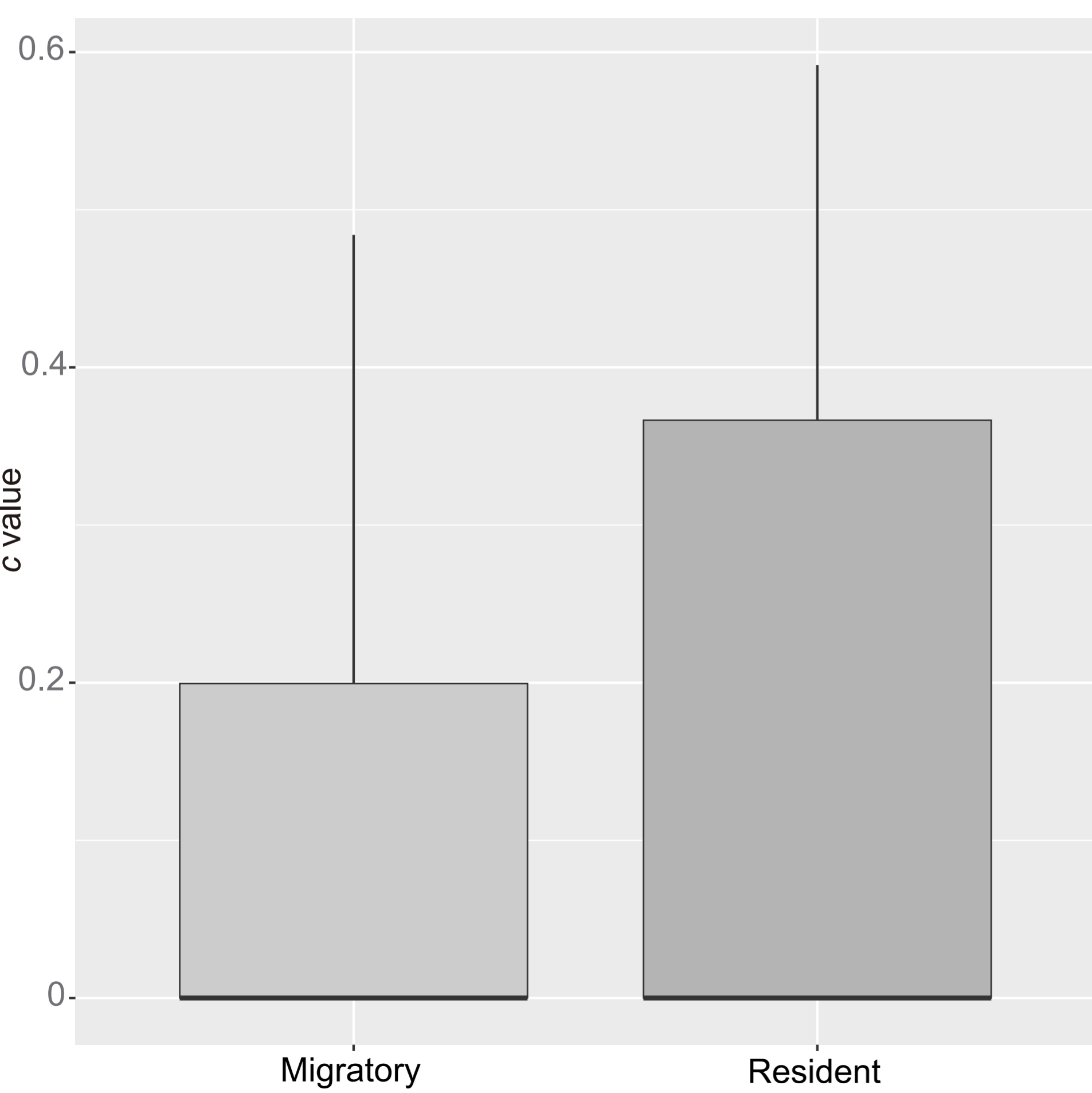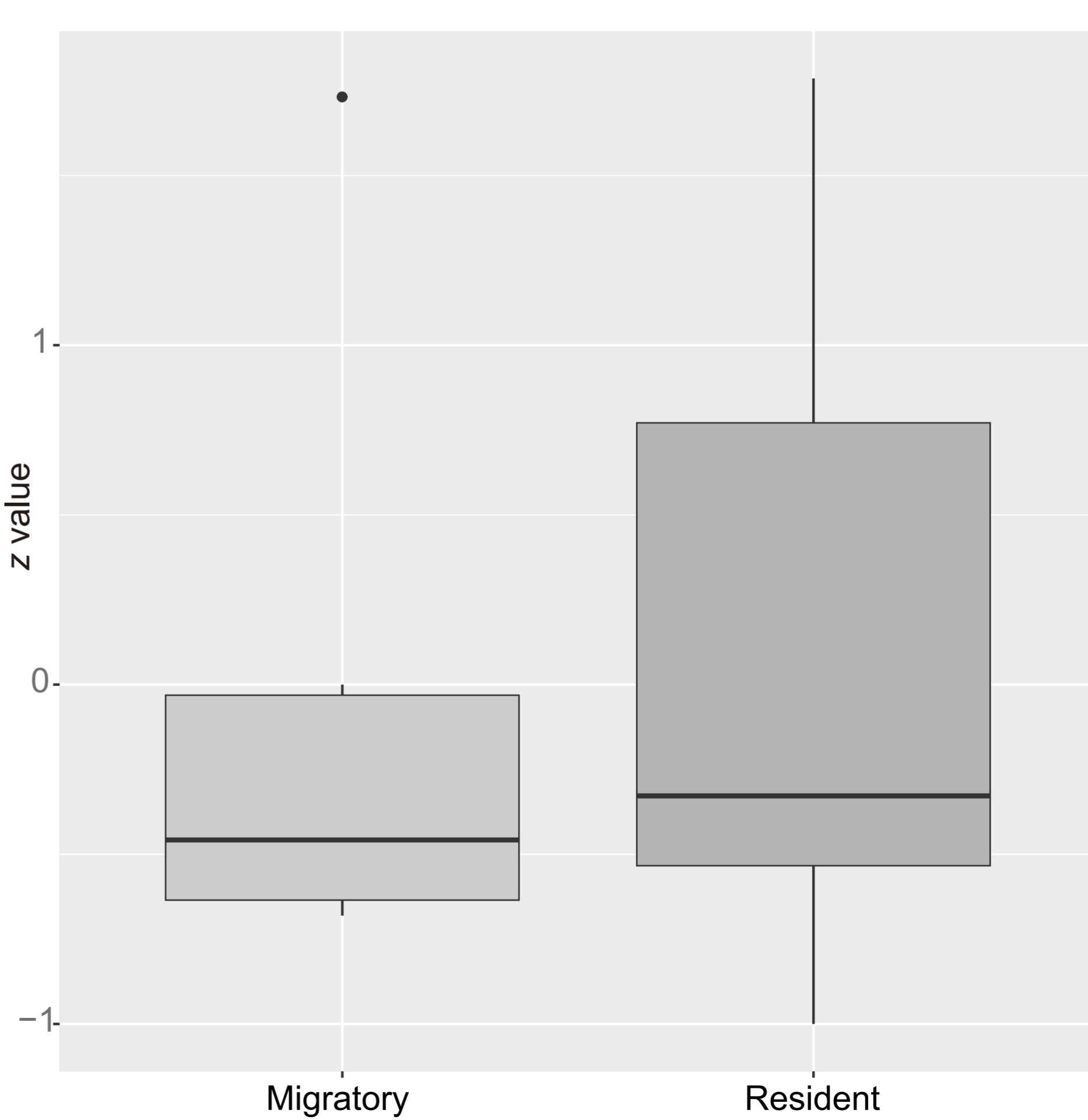

Supplement: Supplementary file 3 — Figure S1 [file ECE3-10-8579-s003.pdf]

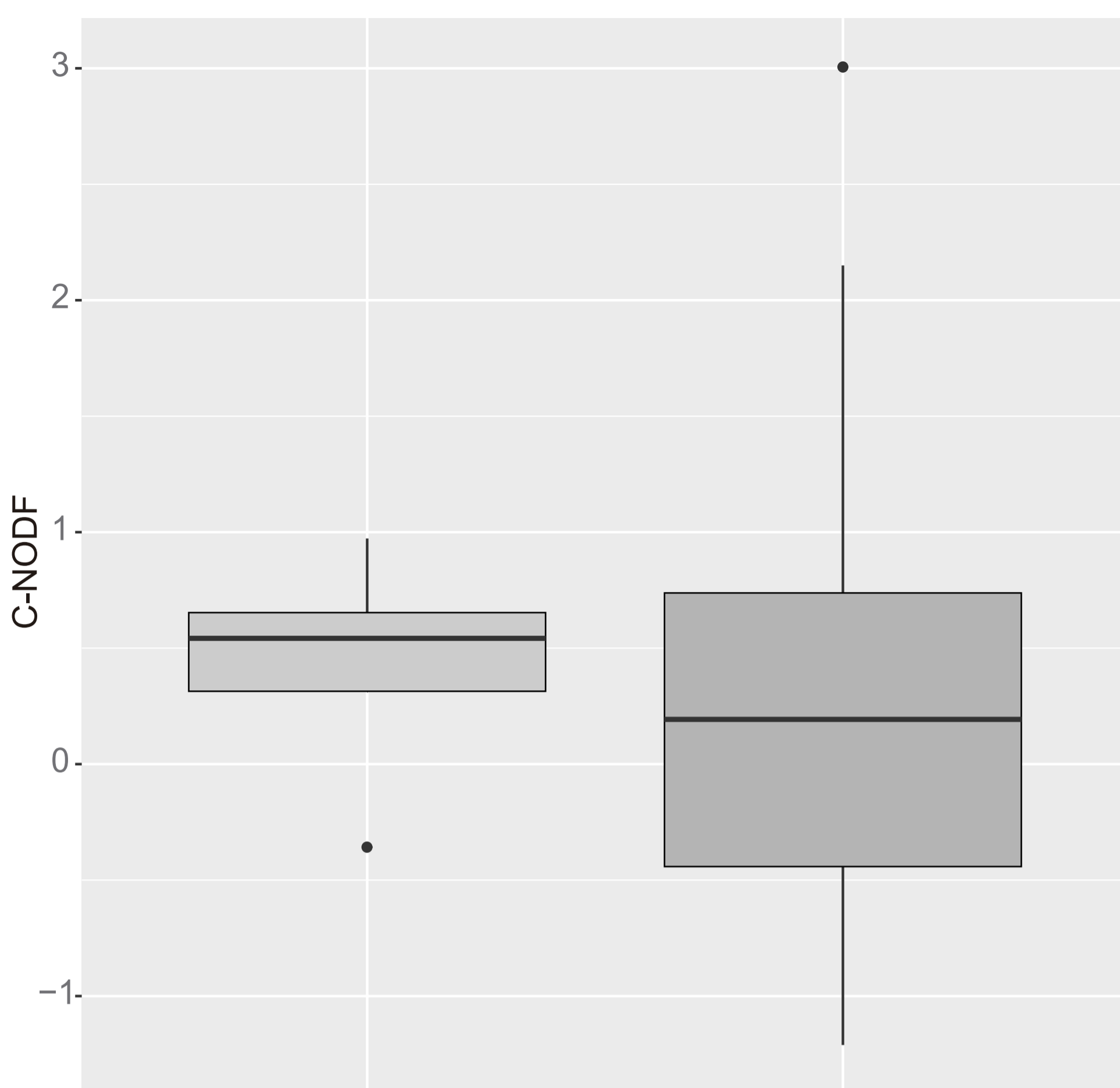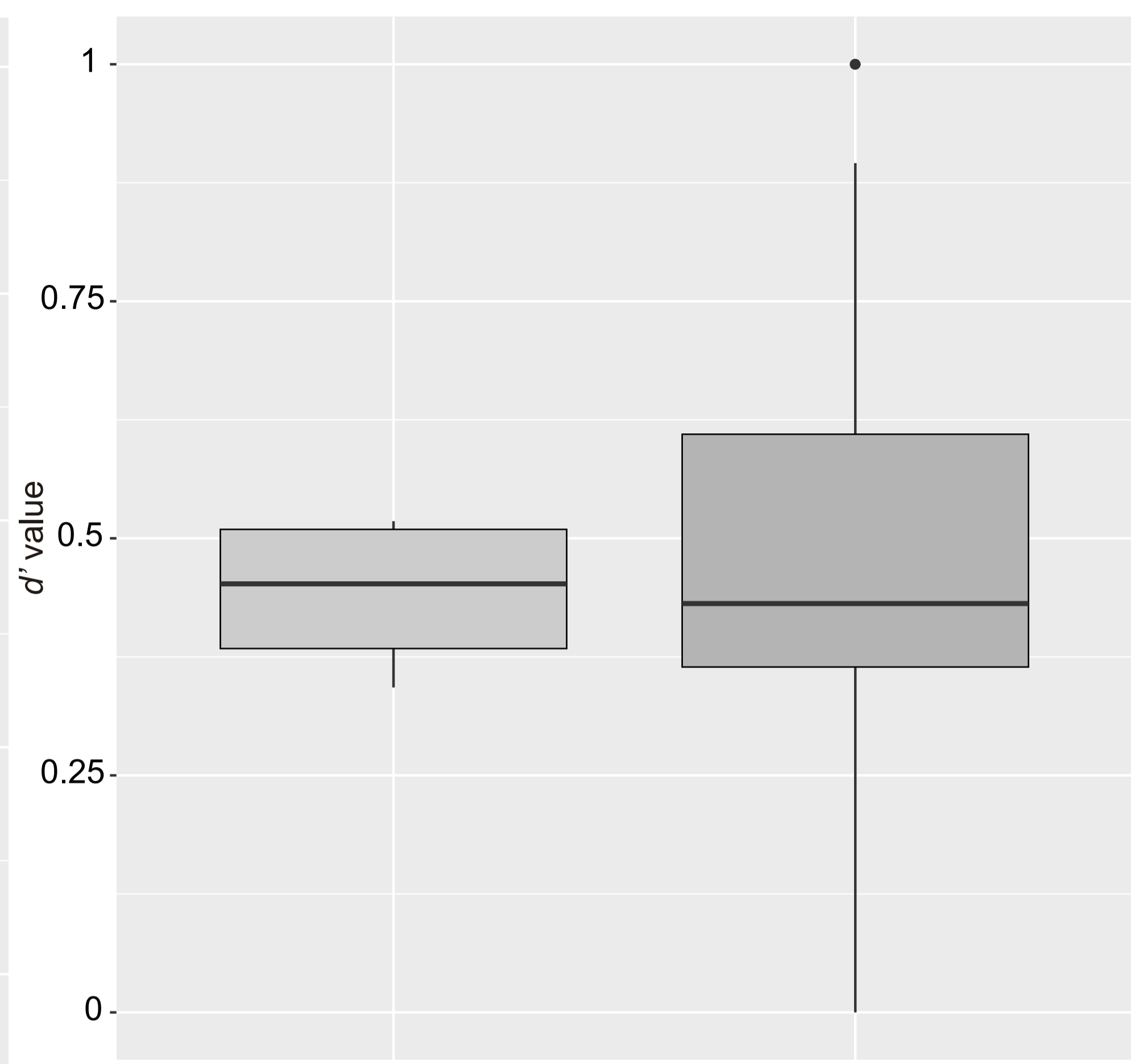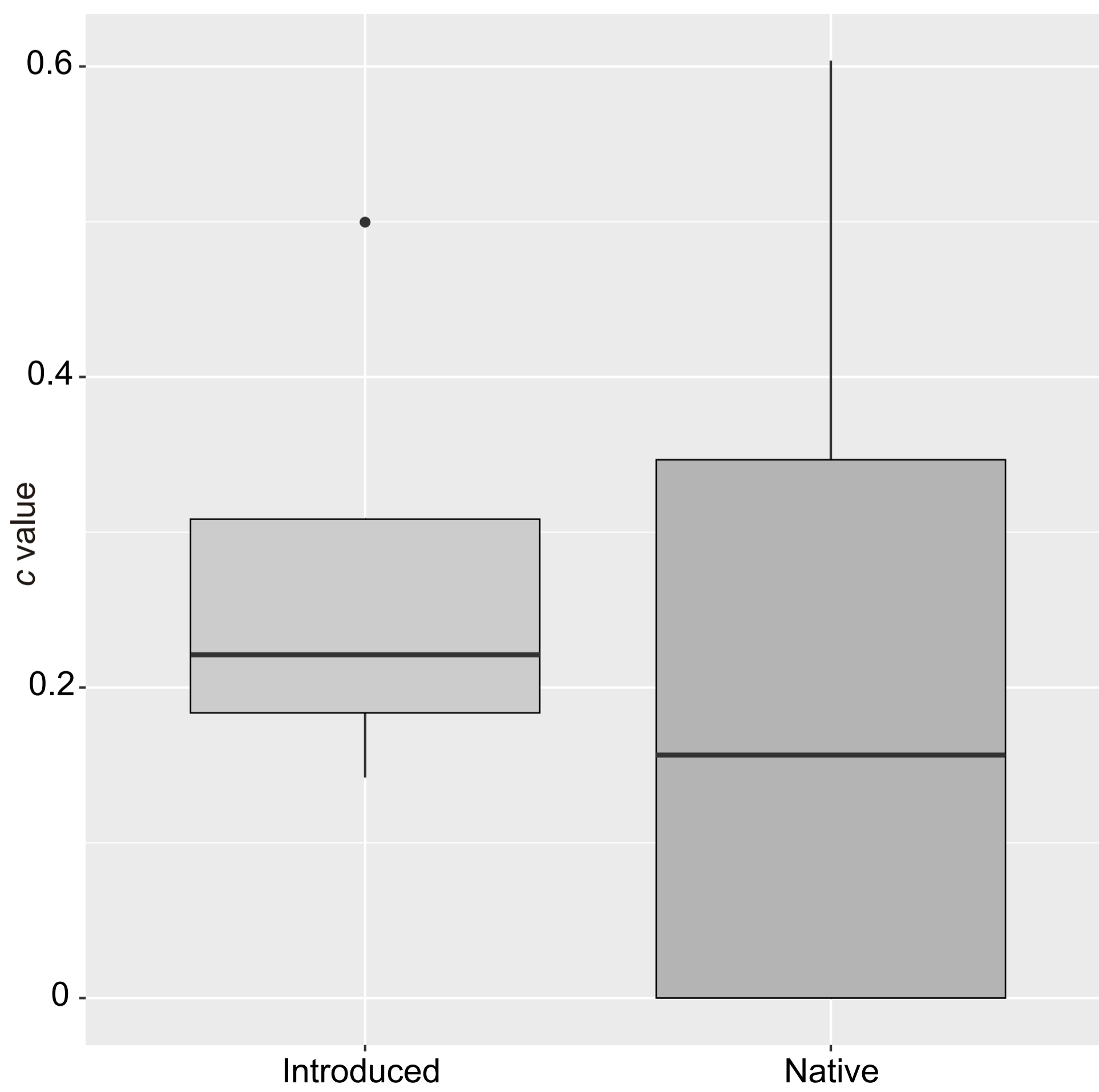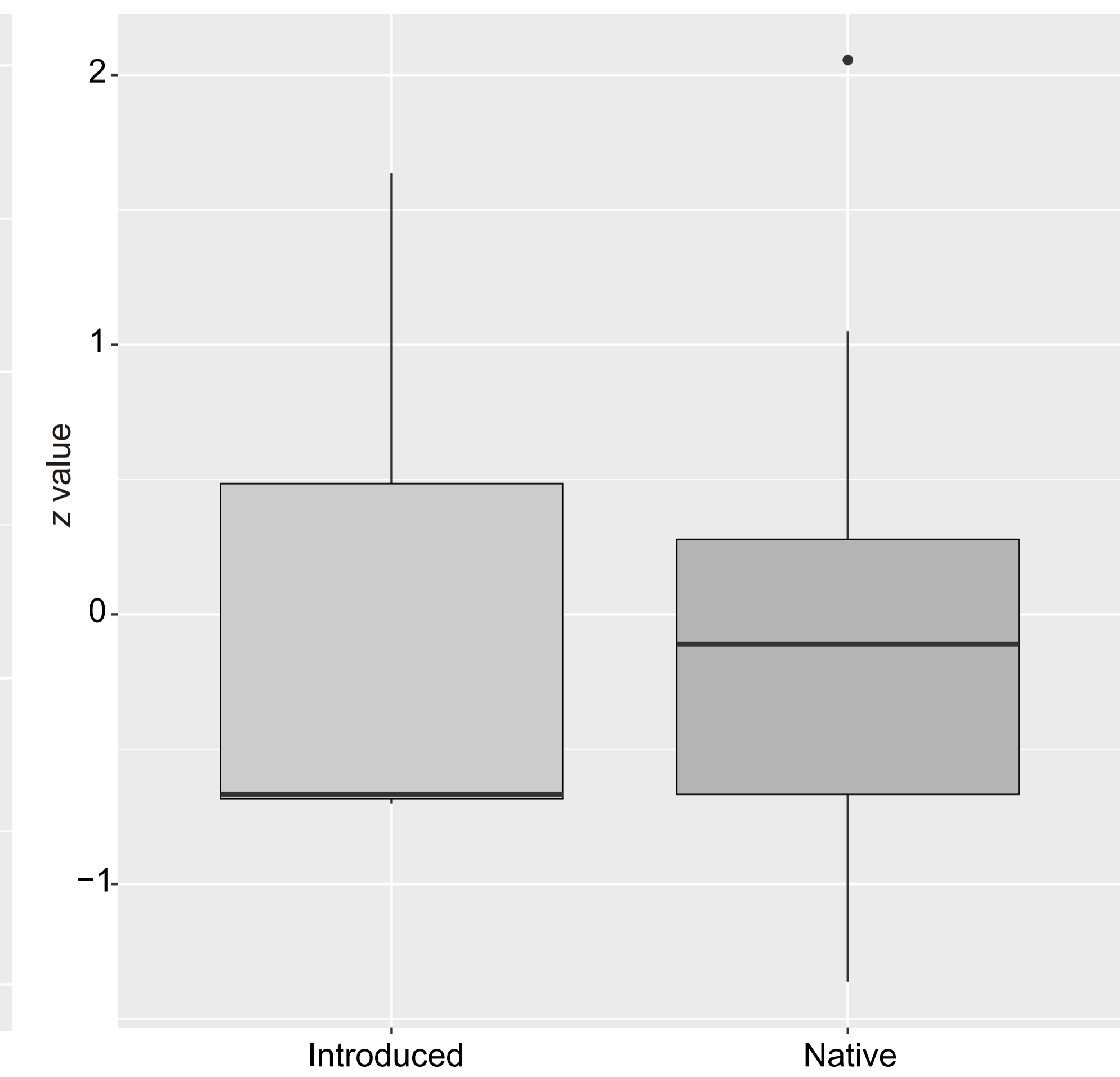

Supplement: Supplementary file 4 — Figure S2 [file ECE3-10-8579-s004.pdf]
